# Macroeconomic fluctuations and the prioritization of healthcare funding by local governments: longitudinal evidence from 5461 Brazilian municipalities

**DOI:** 10.1093/heapol/czag043

**Published:** 2026-03-30

**Authors:** Helena Arruda, Karen Codazzi, Rudi Rocha, Marc Suhrcke, Thomas Hone

**Affiliations:** Instituto de Estudos para Políticas de Saúde (IEPS), São Paulo, Brazil; Instituto de Estudos para Políticas de Saúde (IEPS), São Paulo, Brazil; São Paulo School of Business Administration, Fundação Getulio Vargas (FGV), São Paulo, Brazil; Instituto de Estudos para Políticas de Saúde (IEPS), São Paulo, Brazil; São Paulo School of Business Administration, Fundação Getulio Vargas (FGV), São Paulo, Brazil; Luxembourg Institute of Socio-economic Research (LISER), Esch-sur-Alzette, Luxembourg; Centre for Health Economics, University of York, York, United Kingdom; Instituto de Estudos para Políticas de Saúde (IEPS), São Paulo, Brazil; Public Health Policy Evaluation Unit, School of Public Health, Imperial College London, London, United Kingdom

**Keywords:** health systems financing, macroeconomic fluctuations, Brazil, healthcare spending

## Abstract

Securing sufficient healthcare funding remains a critical challenge for many low- and middle-income countries (LMICs), especially during periods of economic instability. While economic growth often boosts health system finances in LMICs, little is known about how local governments adjust healthcare spending in response to macroeconomic fluctuations, particularly during recessions. This study analyses a longitudinal dataset of 5461 Brazilian municipalities between 2004 and 2017, using fixed-effect panel regressions to examine the relationship between municipal Gross Domestic Product (GDP) changes and healthcare spending. There is a positive elasticity between the economic conditions, measured by GDP, and municipal financial expenditures, particularly health and social expenditures. A 1% increase in municipal GDP per capita was associated with a 0.06% [95% confidence interval (CI) 0.04–0.09] increase in total municipal expenditures and a 0.12% (95% CI 0.06–0.18) increase in health and sanitation expenditures. However, during recessions, municipal governments reduced health expenditures, particularly for capital investments. Municipalities with lower income per capita and lower private insurance coverage were more vulnerable to these cuts in economic downturns. We find suggestive evidence that the cuts in poorer municipalities and in municipalities relying disproportionately on federal transfers were more pronounced for social and health expenditures, although the difference between municipalities is small. These findings highlight the sensitivity of local finances to economic fluctuations, underscoring the need for policies that protect healthcare funding during economic downturns.

Key messages

- Economic growth is linked to healthcare spending across Brazilian municipalities, and economic downturns lead to significant cuts in healthcare budgets, particularly in human resource expenditures.
- Poorer municipalities, those with lower private insurance coverage, and greater dependence on federal and state resources are more susceptible to budget cuts during economic downturns, highlighting their fiscal vulnerability.
- Healthcare financing in low- and middle-income countries is vulnerable to economic fluctuations, especially during recessions, posing a critical challenge for local governments.

## Introduction

Securing adequate and sustainable financing for healthcare is a major challenge for health systems in low- and middle-income countries (LMICs; World Health Organization 2015, McIntyre and Kutzin 2016, Behera and Dash 2019a, 2019b). Adequate funding is essential for delivering high-quality accessible services, improving population health outcomes, and protecting households from catastrophic healthcare costs. Furthermore, there will be an increased demand for health spending in these countries in the next decades. In Brazil, e.g. projections indicate that public healthcare spending needs to increase from <4% to ∼5% of the national Gross Domestic Product (GDP) by 2045 (Rocha et al. 2021). However, in LMICs, health system financing is often fragile, vulnerable to shocks such as economic crises and political instability (Mladovsky et al. 2012, Witter et al. 2020). Understanding how macroeconomic shocks influence fiscal resource allocation decisions is critical to strengthening health system resilience.

The concept of Domestic Fiscal Space for Health (DFSH)—a government’s capacity to allocate sufficient resources for health without jeopardizing fiscal sustainability (Heller 2005, Tandon and Cashin 2010)—provides a useful framework for this analysis. Macroeconomic conditions strongly shape DFSH by affecting government revenues and health spending priorities alongside other factors, such as tax collection efficiency, budgetary prioritization of health, and allocative efficiency (Velenyi and Smitz 2014, Barroy and Gupta 2021). While economic growth generally increases healthcare expenditures, economic downturns often constrain public budgets, forcing difficult trade-offs. However, the extent to which healthcare spending is protected or cut relative to other sectors during economic recessions is poorly understood, particularly in LMICs.

Although multiple studies have examined the elasticity of GDP and public health expenditure, most studies focus on high-income countries (Gerdtham and Jonsson 2000, Xu et al. 2011, Younsi et al. 2016, Zhou et al. 2020). These studies often overlook the institutional and fiscal constraints of LMICs (Behera and Dash 2019b) and frequently rely on cross-sectional analysis that fails to capture the dynamic nature of public budgeting. Furthermore, there is little evidence on how economic fluctuations impact healthcare spending at the subnational level within countries (Giannoni and Hitiris 2002, Fedeli 2015, Behera and Dash 2019a). In decentralized systems, like Brazil’s Unified Health System (Sistema Único de Saúde (SUS)), local governments play a central role in healthcare financing and are therefore directly exposed to local economic fluctuations. Because municipal economies may experience shocks that diverge from national trends, localized downturns can affect local sources of health financing. About one-third of municipalities in Brazil are in recession in any given year, though this share varies substantially and reaches nearly two-thirds in some years analysed in this research.

Brazil presents an ideal setting to explore these questions due to its extensive municipal-level data and its decentralized healthcare system, which was established following the 1988 Constitution and the creation of SUS in 1990 (Castro et al. 2019). Municipal governments in Brazil shoulder a substantial portion of healthcare financing and delivery, and they are often dependent on additional resources via transfers from state and federal levels (Menezes et al. 2019). Public health financing in Brazil is shaped by a set of constitutional and infra-constitutional fiscal rules that define both minimum spending requirements and the allocation of responsibilities across federal, state, and municipal governments. Since the creation of the SUS, health has been established as a shared responsibility across levels of government, with decentralized service provision and co-financing as core principles (Piola et al. 2013).

A central pillar of Brazil’s health financing framework is the system of earmarked minimum expenditures. Constitutional Amendment 29 (Emenda Constitucional (EC) 29) of 2000 required states and municipalities to allocate at least 12% and 15% of their own revenues to health, respectively, while federal spending was initially indexed to the previous year’s expenditure adjusted by GDP growth (Clarke et al. 2024). Municipal own-source revenues include taxes, such as the services tax (Imposto Sobre Serviços (ISS)), the urban property tax (Imposto sobre a Propriedade Predial e Territorial Urbana (IPTU)), and the real-estate transfer tax (Imposto sobre Transmissão de Bens Imóveis (ITBI)), complemented by constitutionally mandated intergovernmental transfers distributed according to population-based criteria that favour smaller municipalities.

These rules were later consolidated by Complementary Law 141 (Lei Complementar (LC) 141) in 2012, strengthening enforcement and standardizing accounting practices. As a result, municipalities, which are responsible for most primary care and an increasing share of hospital services, became structurally committed to financing health expenditures from their own revenues, making local health spending closely tied to municipal fiscal capacity (Cruz et al. 2022).

At the federal level, fiscal rules changed after 2015. Constitutional Amendment 86 linked minimum health spending to net current revenue but was quickly replaced by Constitutional Amendment 95 (2016), which introduced a binding real expenditure ceiling. This reform decoupled federal health spending from economic and demographic growth, while states and municipalities remained subject to minimum spending requirements, further shifting the financing burden towards subnational governments.

Within the SUS, municipalities are primarily responsible for executing and managing health services, as established by the 1988 Constitution and Law No. 8080/1990. Municipal governments deliver health actions through local health departments, with a central role in primary care—such as basic health units and family health teams—and surveillance activities. Higher-cost services rely more on state and federal financing, so adverse local economic shocks that reduce municipal tax revenues can directly affect health service delivery, with limited short-run substitution.

Understanding the fiscal vulnerability of local health systems to economic fluctuations serves to inform policies aimed at buffering healthcare financing from recessions and leveraging health system resilience, not least because health spending matters for health outcomes in LMICs (Bokhari et al. 2007, Farag et al. 2013, Clarke et al. 2024). Our study provides evidence from Brazil’s decentralized health system and offers insights into how local governments in LMICs can protect healthcare financing during periods of economic uncertainty. This study uses a panel dataset covering 5461 Brazilian municipalities from 2004 to 2017 to examine how local macroeconomic fluctuations affect healthcare financing. We investigate the impact of changes in municipal GDP per capita on healthcare spending and assess whether health budgets are disproportionately affected during recessions. We also analyse how municipal characteristics—such as income per capita levels, private health insurance coverage, and dependence on federal transfers—influence these relationships.

## Materials and methods

### Study design

This study is a longitudinal panel data analysis of 5461 Brazilian municipalities, covering the period from 2004 to 2017. The analysis includes all municipalities except those created during the period or with >5 years of missing key data, totalling 109 exclusions out of 5570 municipalities. The time frame begins in 2004 to accommodate the transition of new financing rules for municipal governments introduced in 2000 and ends in 2017 to exclude the confounding impact of the coronavirus disease 2019 (COVID-19) pandemic. We implement a two-way fixed-effects regression approach to investigate the relationship between fluctuations in GDP per capita at the municipal level and various types of budgetary measures, with a particular focus on social and health expenditures. The unit of analysis is the municipality, which is the smallest administrative entity in Brazil. Municipal governments are primarily responsible for health service financing and delivery, which makes them particularly sensitive to local economic fluctuation. The use of a fixed-effects model allows us to control for unobserved, time-invariant characteristics at the municipality level, such as geography or historical differences in healthcare infrastructure, that could otherwise bias the results. Year fixed-effects account for nationwide shocks or trends that uniformly affect all municipalities, while municipality-specific linear and quadratic time trends are also included to control for non-linear local variations in economic activity over time.

### Data

All data used in this analysis are publicly available from official sources. Municipal revenue and expenditure data were obtained from the Accounting and Fiscal Information System of the Brazilian Public Sector (Finbra; Tesouro Nacional 2024), an information system from the Brazilian Ministry of Finance that provides yearly data on municipal revenues and expenditures by spending category. Specific health budget data were sourced from the Information System on Public Health Budgets (Ministry of Health of Brazil 2024b), which specifically monitors health-related expenditures at the municipal level. The National Registry of Health Establishments provided information on the number of municipal hospitals (Ministry of Health of Brazil 2024a), while data on private health insurance coverage came from the National Agency of Supplementary Health (Agência Nacional de Saúde Suplementar 2024). Population, municipal GDP, and inflation-adjusted indices (Índice Nacional de Preços ao Consumidor Amplo (IPCA)) were provided by the Brazilian Institute of Geography and Statistics (Instituto Brasileiro de Geografia e Estatística (IBGE) 2016).

### Variables

The primary dependent variables include total municipal expenditures, total revenues, and social expenditures (a subcategory of total expenditures that includes spending on health, education, social assistance, transportation, urbanization, and culture). Non-social expenditures, like public security, judiciary and administrative expenditures, comprise all other components of total expenditures not classified as social expenditures. An important outcome of interest is health expenditures, which are a subset of social expenditures and also include expenditures on sanitation. Health expenditures are further disaggregated according to their funding source. Specific categories of health spending (such as personnel expenditure and investment in health) were also analysed.

The primary independent variable of interest is the annual GDP per capita at the municipal level, capturing local macroeconomic conditions. We also created a binary variable indicating whether a recession occurred in a given municipality and year, defined as a decline in GDP per capita from year *t* − 1 to year *t*. Recessions were further classified into more severe or less severe, depending on whether the decline in GDP per capita exceeded the median drop across all recession periods in the sample.

For the heterogeneity analysis, we used household income per capita, private health insurance coverage rates (i.e. the proportion of the population covered by private insurance), and the share of municipal health expenditures funded by own resources to split the sample into subgroups. All monetary variables, including revenue, expenditures, and GDP, were expressed in per capita values using population by municipality and deflated to 2022 current prices using the IPCA index. We used the logarithmic transformation of these per capita values to facilitate interpretation of the regression coefficients in terms of elasticities. Additionally, for international comparability, all monetary results were converted to US dollars using the Brazilian Central Bank conversion rates on 20 September 2024.

To assess the impacts of cyclical fluctuations in economic conditions, which is the focus of our analysis, we de-trended the GDP and budget variables using a Hodrick–Prescott (HP) filter, which is commonly used in the analysis of macroeconomic fluctuations (Ionides et al. 2013, Avendano et al. 2017, Hone et al. 2019). This method isolates the cyclical component of GDP, removing long-term trends and allowing us to focus on short-term economic variations. Linear interpolation was used to fill in missing data for up to 5 consecutive years for outcomes and independent variables at the municipality level. About 29.5% of municipalities had some missing observations (between 1 and 4) in the key variables, and these gaps were filled through interpolation. Further details on the variables and data sources are provided in Supplementary Table A.1.

### Statistical analysis

The empirical approach builds on widely used models aimed at assessing the impacts of economic fluctuations on public budgets and health outcomes (Ruhm 2016, Hollingsworth et al. 2017, Hone et al. 2019). The general estimating equation is as follows:


(1) $$\begin{array}{rl} \log(Y_{mt}) & =\beta_{1}*\log(GDP_{m(t-2)})+\beta_{2}\log(GDP_{m(t-1)})+\beta_{3}\log(GDP_{mt})\\ & \;+\beta_{4}\log(GDP_{m(t+1)})+\beta_{5}\log(GDP_{m(t+2)})+\mu_{m}\\ & \;+\sigma_{t}+\gamma_{1}t_{m}+\gamma_{2}t_{m}^{2}+\varepsilon_{mt}, \end{array}$$

where *Y_mt_* represents the municipal budget variable of interest in municipality *m* and year *t*; *GDP_mt_* is the GDP per capita in municipality *i* in year *t*; *µ_m_* represents municipality fixed effects; *σ_t_* refers to year fixed effects, while *t_m_* and $t_{m}^{2}$ are linear and squared time trends specific to municipality *m*. Linear and quadratic time trends by municipality are included to absorb long-term municipal-specific trends in economic growth. The log–log specification implies that the coefficients on GDP per capita can be interpreted as elasticities, capturing the percentage change in municipal revenues or expenditures associated with a 1% change in local GDP per capita.

We include two lags and two leads of GDP per capita to examine dynamic expenditure elasticities, allowing municipal budget responses to vary over time around economic fluctuations. Regressions were weighted by municipal population to account for heterogeneity in population size. Finally, *ɛ_mt_* is the error term. Standard errors are clustered at the municipality level to allow for serial correlation within municipalities. By assuming that de-trended GDP per capita variation, conditional on fixed effects and municipal-specific time trends, is exogenous and unforeseen by policymakers, we can interpret our findings as causal estimates.

We used equation 1 to analyse the impact of macroeconomic conditions on total revenues, total expenditures, and broad expenditure categories, including social, non-social, health, and education expenditures. We then focused on health expenditures, further disaggregating them into two specific components: personnel (human resource, HR) and investment expenditures.

We further examined the impact of recessions on the same outcomes described above by replacing the continuous GDP variable with a binary variable indicating the occurrence of a recession in a given year. The recession indicator does not represent elasticity *per se*, but rather a semi-elasticity capturing discrete changes in expenditures associated with downturn episodes. To explore relevant heterogeneities, we investigated how the relationship between recessions and expenditure outcomes varied across different types of municipalities by dividing them into three key groups. First, we categorized municipalities according to their per capita household income levels. Second, we split the sample by private health insurance coverage, distinguishing municipalities with coverage above or below the median share of beneficiaries. Finally, we compared municipalities based on their dependence on own-source health funding. Since this variable fluctuates considerably over time, we calculated the mean share of own-source health spending across all years for each municipality and used the sample median as the cut-off to classify municipalities into higher or lower dependence groups.

## Results

**Figure 1:**
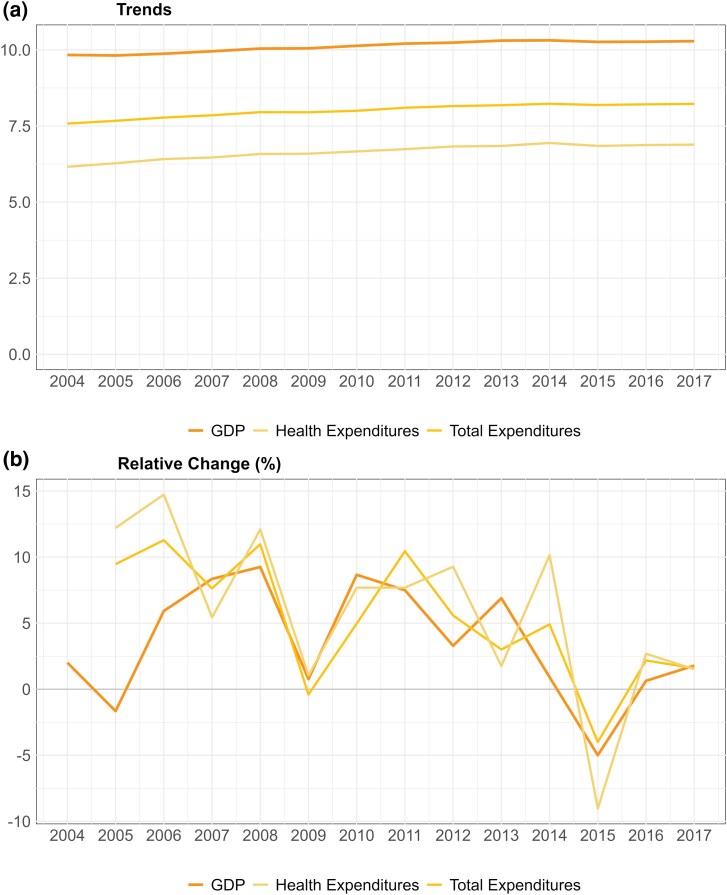
Trends and relative changes in mean municipal GDP per capita and health expenditure per capita (2004–17). The figure shows the evolution of municipal trends (a) and relative changes of GDP per capita (b) and is deflated using IPCA.

Our sample included 5461 municipalities each year, covering the period 2004–17 (Table 1). Mean municipal GDP per capita was R$30 436 (US$5556). The average household income per capita in 2010 was R$1069 (US$195.2), whilst the mean municipal population was 34 000 inhabitants. The mean municipality coverage with private health insurance was 7.6%. The mean annual municipal revenue was R$3781.1 (US$690) per capita and expenditures were R$3060 (US$558) per capita. Most municipal expenditures (80%) were allocated to social categories including health and sanitation, social assistance, transportation, urbanization, education, and culture. Health expenditures alone accounted for ∼25% of total municipal spending, on average, predominantly financed by municipalities’ own resources (69% on average). We observe increasing trends for GDP per capita (mean annual change in GDP was 5%) and mean municipal expenditures per capita (both for total and specifically for health, with mean annual increases of 5.1% and 5.7%, respectively). Annual changes in municipal expenditures and GDP per capita were generally correlated over time (Fig. 1).

**Table 1:** Descriptive statistics of the budget variables used in the model.

	Q1	Median	Mean	Q3	SD
Municipal budgetary variables (R$ at 2022 prices, per capita)					
GDP	10 416.3	18 075.2	25 049.4	30 436.5	1 328 757.5
Change in GDP	−361.9	507.5	789.2	1649.0	551 521.4
Income	625.4	1024.2	1069.7	1396.4	502.5
Total revenue	2498.9	3266.4	3781.1	4450.9	2079.7
Total expenditures	2070.0	2702.6	3060.6	3616.0	1555.3
Social expenditures	1747.9	2279.4	2530.4	2988.9	1510.4
Non-social expenditures	519.2	747.0	945.5	1126.5	803.0
Health expenditures	518.5	701.2	793.9	961.7	412.8
Health expenditures—municipal resources	270.7	414.7	510.2	645.8	348.6
Health expenditures—federal/state resources	179.7	254.7	284.8	351.1	169.7
Health HR expenditures	245.6	346.5	392.8	480.6	224.9
Health investment expenditures	10.9	26.5	46.8	57.6	65.6
Other variables					
Relative change in GDP (%)	−2.2	3.9	5.1	10.4	5593.7
Population	5341	11 150	34 681	23 881	12 099 887
Privately insured individuals (%)	0	3.1	7.6	9.6	10.9

The table provides five key statistical measures: the first quartile (Q1), median, mean, third quartile (Q3), and standard deviation (SD) for each budget category. GDP, Gross Domestic Product; HR, human resources.

**Figure 2:**
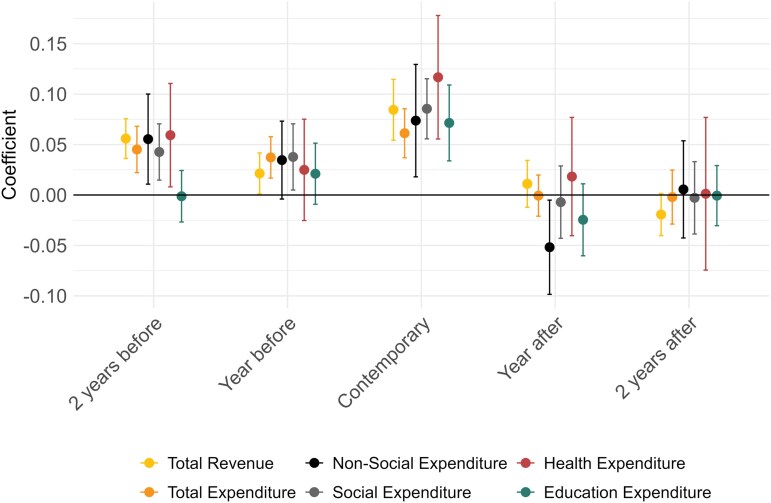
Dynamic effects of GDP fluctuations on municipal revenues and expenditures. This figure shows graphically results from equation 1, which is based on a municipality-by-month panel for the 2004–17 period. The dependent variables refer to total municipal revenue and expenditures both total and split by categories. Regression results appear at Supplementary Table A.2.

Figure 2 reports the estimated elasticities of municipal revenues and expenditures with respect to municipal GDP per capita, based on the two-way fixed-effects panel regression model (see Supplementary Table A.2 for further information). There was a general pro-cyclical association between GDP per capita and municipal government revenues and expenditures. A 1% increase in current GDP per capita was associated with a 0.084% [95% confidence interval (CI) 0.05–0.11] increase in total municipal revenue per capita, a 0.06% (95% CI 0.04–0.09) increase in total expenditure per capita, a 0.09% (95% CI 0.06–0.12) increase in social expenditure per capita, a 0.07% (95% CI 0.02–0.13), and a 0.07% (95% CI 0.02–0.13) increase in non-social expenditures per capita. Notably, municipal health expenditure displays the highest elasticity with respect to GDP per capita [0.12% (95% CI 0.06–0.18)], suggesting that health spending is particularly responsive to local economic conditions. We also document dynamic responses, with lagged associations between past GDP per capita and current revenues and expenditures. For example, a 1% increase in GDP in the previous year was associated with a 0.02% (95% CI 0.00–0.04) rise in current total revenue, a 0.04% (95% CI 0.02–0.06) increase in current total expenditures, a 0.04% (95% CI 0.00–0.07) increase in current social, and a 0.03% (95% CI 0.00–0.07) increase in current non-social expenditures per capita.

**Figure 3:**
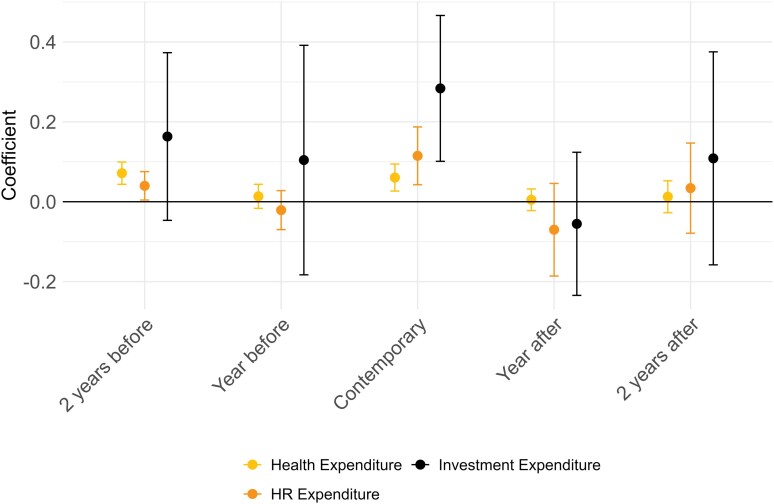
Dynamic effects of GDP fluctuations on municipalities total health expenditure, investment health expenditure, and HR health expenditures. This figure shows graphically results from equation 1, which is based on a municipality-by-month panel for the 2004–17 period. The dependent variables refer to total health spending, investment in health expenditure and HR health expenditures. Regression results appear at Supplementary Table A.3.

**Figure 4:**
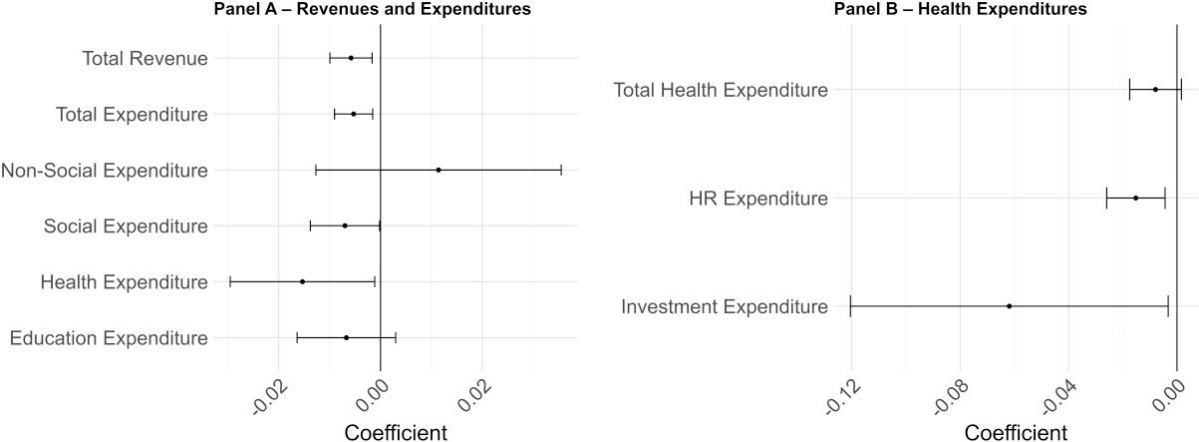
Recession effects on revenues and expenditures, total and by categories. This figure shows graphically results from an adaptation of equation 1 where we replace the continuous GDP variable with a binary variable indicating the occurrence of a recession in a given year. The dependent variables in (a) refer to total municipal revenue, and expenditures both total and split by categories. On (b), dependent variables refer to health spending, including total health spending, and specific categories (HR and investments). Regression results appear at Supplementary Tables A.4 and A.5.

In Fig. 3 (and Supplementary Table A.3), we examine the elasticity of municipal health expenditures with respect to local GDP, distinguishing between total health spending, spending on HR, and investment expenditures. Changes in GDP per capita are positively associated with all categories of health spending. A 1% increase in GDP per capita was associated with a 0.06% (95% CI 0.03–0.09) increase in overall health expenditures per capita and a 0.11% (95% CI 0.04–0.19) increase in expenditures on HR in health. Investment expenditures exhibit a substantially higher elasticity with respect to GDP per capita, a 1% increase in GDP per capita in a given current year was associated with a 0.28% increase (95% CI 0.1–0.47) in investments. Interacting GDP with boom and recession indicators, we find no statistically significant differences in fiscal elasticities, although point estimates suggest stronger revenue and, to a lesser extent, health and health workforce responses during downturns (Supplementary Figures A1 and A2). We examined the specific association of revenues and expenditures with recession (Fig. 4 and Supplementary Tables A.4 and A.5). Recessions were negatively associated with revenues and expenditures, especially health and social categories of expenditure. Compared with periods of growth or stability, having a recession in a given year was associated with a reduction in total revenue per capita of −0.58% (95% CI −0.99 to −0.17) and in total expenditure per capita of −0.53% (95% CI −0.90 to −0.15). Social expenditure per capita decreased by −0.70% (95% CI −1.38 to −0.00), and health expenditure per capita decreased by 1.53% (95% CI −2.95 to −0.11). Non-social expenditure per capita was not significantly affected by recessions. When disaggregating health expenditures, recessions are associated with particularly large contractions in investment spending [about −6.19% (95% CI −12.05 to −0.32)].

**Figure 5:**
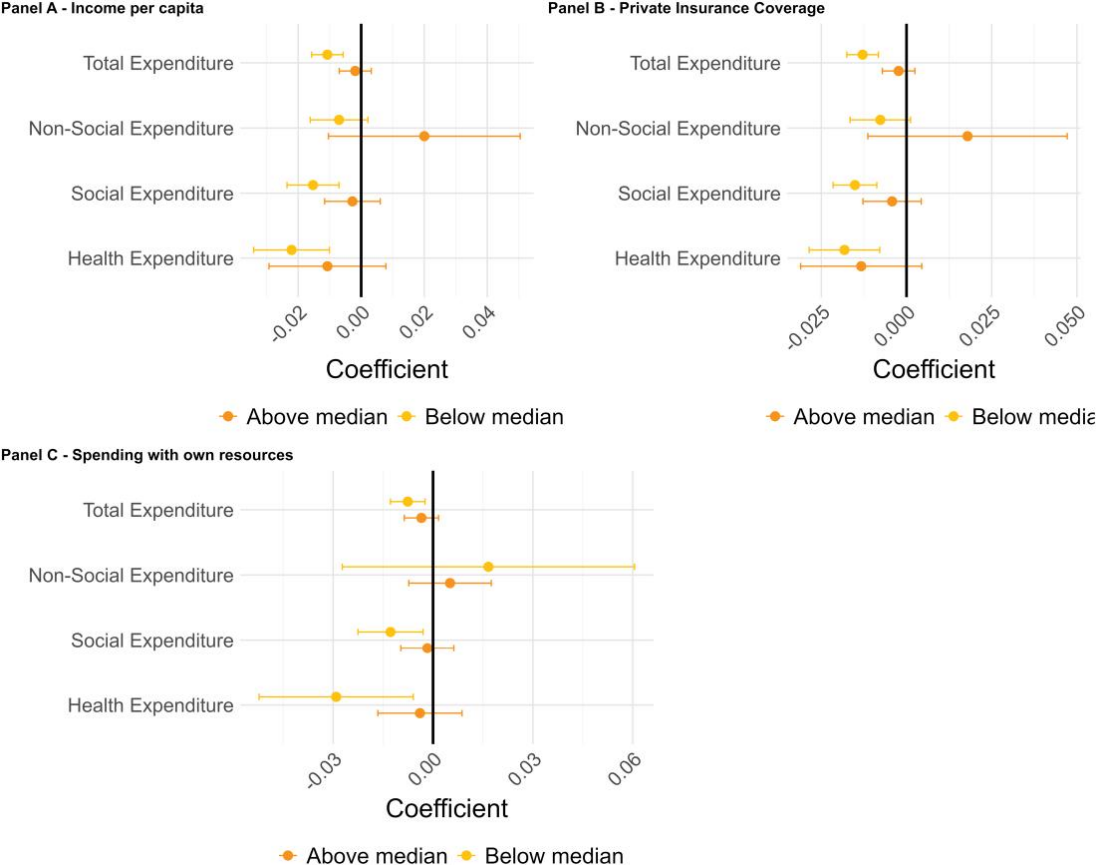
Heterogeneous recession effects on expenditures. This figure shows graphically results from an adaptation of equation 1 where we replace the continuous GDP variable with a binary variable indicating the occurrence of a recession in a given year. In (a), we split municipalities according to whether they are above or below the median income per capita in 2010 (approximately the middle of our sample). In (b), we split municipalities according to whether they are above or below the median private insurance coverage, considering the average coverage in our sample of years. In (c), we split municipalities according to whether they are above or below the median in the levels of municipal health spending with their own resources, considering the average spending in our sample of years. Dependent variables refer to total expenditures and split by categories. Regression results appear at Supplementary Tables A.6–A.8.

We further investigate heterogeneity in these associations across municipalities (Fig. 5 and Supplementary Tables A.6–A.8). First, we explored whether the relationship between economic recessions and municipal expenditures varied with municipal household income per capita levels. The results provide suggestive evidence that the association between recession and reductions in expenditures was stronger in poorer municipalities (i.e. where average income per capita in 2010 was below the median), although the differences between income groups are relatively small, particularly for health expenditures. In municipalities below the median income, recessions were associated with a reduction in total expenditures of −1.07% (95% CI −1.57 to −0.57), and a reduction in social expenditures of −1.53% (95% CI −2.35 to −1.70). For municipalities above the median income, changes in expenditures associated with recessions were not statistically significant: −0.19% (95% CI −0.70 to 0.32) for total expenditures and −0.28% (95% CI −1.16 to 0.61) for social expenditures.

Secondly, we compared municipalities with high and low private health insurance coverage as a proxy to private spending since, given data limitations, we cannot include out-of-pocket spending. In municipalities where private insurance coverage was below the median, recessions were associated with a larger reduction in total expenditures, estimated at −1.29% (95% CI −1.75 to −0.83), while municipalities above the median experienced a smaller, non-significant decline of −0.23% (95% CI −0.71 to 0.25). A similar pattern was observed for social expenditures: in municipalities below the median, recessions were associated with reductions of −1.51% (95% CI −2.15 to −0.87), whereas in those above the median, the estimated reduction was −0.42% (95% CI −1.28 to 0.43). However, the difference between these groups was small and not statistically significant.

Finally, we categorized municipalities based on their reliance on using their own resources for health expenditures, relative to other funding sources. Municipalities more reliant on state and federal transfers (below the median share of own resource revenues) exhibit larger expenditure reductions associated with recessions for both social and health expenditures. In below-median municipalities, recessions were associated with an average decline of −1.28% (95% CI −2.25 to −0.30) in social spending, while above-median municipalities exhibit a smaller coefficient of −0.17% (95% CI −0.97 to 0.13). When we restrict the analysis to health expenditures, in municipalities below the median, recessions were associated with a reduction in health spending of −2.91% (95% CI −5.22 to −0.59), whereas those above the median were associated with a cut of −0.39% (95% CI −1.66 to 0.87). Thus, despite consistent directional patterns, differences across groups are small and statistically insignificant, indicating broadly similar fiscal responses to recessions.

### Sensitivity analyses

We conducted sensitivity analyses by altering the main smoothing parameter of the HP filter from *λ* = 100 to *λ* = 6.25, both of which are standard choices for annual data. While *λ* = 100 produces a smoother trend by filtering out more short-term fluctuations, *λ* = 6.25 allows the trend to follow the data more closely. The results remain qualitatively similar under both specifications, indicating that our findings are robust to reasonable variations in the degree of smoothness imposed by the filter.

We also estimated the models without detrending the GDP and budget variables to compare the results. The results from these additional exercises remained consistent with the main specification presented here.

## Discussion

This study shows that macroeconomic fluctuations significantly affect local government revenues and expenditures in Brazil, particularly on healthcare spending. Economic growth was associated with an increase in spending across various categories, while recessions led to particularly greater reductions in healthcare expenditures, notably in capital investment. We found suggestive evidence that the most vulnerable municipalities—those that are poorer, have lower levels of private health insurance coverage, and rely more on federal and state transfers for health expenditures—were particularly sensitive to recessions and experienced the largest reductions in total and social expenditures.

The pro-cyclicality in health spending that we observe among Brazilian municipalities is in line with previous cross-country studies and the few existing within-country analyses. We found an average elasticity of ∼0.11, which is comparable to 0.18 found for Italian regions (Fedeli 2015) and close to, or within the range of estimates found in cross-country analyses. Other studies have found elasticities of health expenditures ranging from 0.16 to 0.47 across countries (Younsi et al. 2016), a positive relationship between tax revenues and health expenditures ranging from 0.04 to 0.141 across LMICs has been documented (Behera and Dash 2019a, 2019b).

This study found that the elasticities between GDP and expenditure vary across categories of spending, with recessions affecting capital investments in health the most, followed by spending on health personnel. This could be explained by the fact that capital investments are more discretionary and easier to postpone or cancel during fiscal constraints, as they often involve long-term planning and large upfront costs (Bonomo et al. 2021, Richards et al. 2025). In contrast, personnel expenditures are typically more rigid due to contractual obligations and the need to maintain basic service provision (Spilimbergo and Srinivasan 2019, Bonomo et al. 2021), but they may still be affected as governments seek to control recurrent costs under budget pressure. During recessions, per capita health investment spending drops by roughly 6% compared with non-recession years, which is about R$2.9 less per person, given the sample average of R$46.8. For a median-sized municipality (∼11 150 residents), this amounts to ∼R$32 000 fewer reais invested in health infrastructure during a recession year. Although the per capita reduction seems modest, it can force local governments to delay or scale back critical capital projects—e.g. postponing the purchase of new medical equipment or upgrades to health facilities—potentially weakening service delivery capacity. If economic downturns persist or recur, these deferred investments may accumulate and undermine longer-term improvements in local health services and infrastructure.

Our analysis also highlights how municipalities with greater socioeconomic vulnerability (i.e. those with lower per capita income, lower private insurance coverage and greater reliance on external transfers) were the most vulnerable to recessions. In these municipalities, recessions were associated with larger cuts in total, social and health-related expenditures. These effects likely exacerbate existing disparities in public service provision between wealthier and poorer municipalities, as wealthier municipalities appear able to maintain funding and therefore likely service provision levels during recessions. Furthermore, the larger contraction in social spending in poorer municipalities is worrisome, as the populations in these municipalities are likely to be of lower socioeconomic status and more dependent on government-provided services. This disproportionate impact risks deepening health inequalities by reducing access to essential care precisely when economic hardship is greatest. These findings are consistent with the fact that economically vulnerable municipalities often have limited fiscal autonomy and lower capacity to mobilize local resources, making them more susceptible to revenue shortfalls and dependent on intergovernmental transfers (Hansen 2022, Paula and Pinho 2023).

This study has limitations. While we used a robust fixed-effects model to control for unobserved heterogeneity, some potentially relevant factors, such as quality of care indicators, shifting local political priorities, administrative capacity, or unexpected shocks like natural disasters, could not be taken into account due to data constraints. Additionally, while our findings from Brazil offer valuable insights, they may not be fully generalizable to other LMICs, especially those with different health system structures. Future research could extend this analysis to other LMIC contexts, exploring the impact of macroeconomic fluctuations on healthcare financing in different governance and institutional frameworks. Finally, while we control for time-invariant and time-specific unobserved factors, we cannot discard whether non-observable variables affect municipal governments’ decisions about spending. If some local governments react to recessions by smoothing more drastic spending cuts in non-observable ways, for instance, we expect that our estimates represent a lower bound of potential recession impacts.

There are relevant implications for healthcare policy in Brazil and other LMICs. First, municipal governments and their expenditures are affected by recessions and GDP fluctuations. Securing adequate funding for healthcare is a significant challenge, particularly for LMICs, and policymakers should consider strategies to buffer health expenditures from economic downturns. For example, governments may need to create dedicated healthcare stabilization funds to protect critical spending during economic downturns. During the COVID-19 pandemic, many countries created programmes to enhance health system resilience, improve crisis preparedness, and ensure the continuity of essential health services (De Foo et al. 2023, Paschoalotto et al. 2023). The EU4Health programme is one example, focusing on areas such as disease prevention, health promotion, and the digital transformation of health systems (European Commission 2010).

In the Brazilian case, our analysis suggests that there exists vulnerability within the health budget related to spending on personnel and, in particular, capital investments. While capital investment may require large upfront financing, and therefore spending cuts or delays can be rationalized given fiscal constraints, spending reductions in personnel may potentially affect current operations, the delivery of health services and the pressing health needs of the population. Second, targeted financial support may be needed for municipalities that are particularly vulnerable to economic fluctuations. For example, federal and state governments could provide additional funding during recessions to help these municipalities maintain health services and avoid cuts that could negatively impact health outcomes. Finally, building fiscal resilience at the municipal level is essential for safeguarding healthcare financing. Diversifying local revenue sources and improving financial management can help municipalities reduce their reliance on external transfers (Tandon and Cashin 2010). Policymakers could also consider broader economic policies to promote stable and sustainable growth, as economic stability directly influences the fiscal space available for healthcare (Velenyi and Smitz 2014).

The findings may also have broader implications for other LMICs with varying fiscal structures. In more centralized systems, such as in India and China, where the national government controls most health spending, recessions may impact national health budgets or delay funds to local governments. Conversely, in countries like South Africa and Nigeria, with lower private insurance coverage and heavy reliance on external aid, the impact of economic shocks could disproportionately affect vulnerable populations. The Brazilian experience underscores the need for adaptable health financing systems that are resilient to macroeconomic shocks, even in more centralized contexts. Further research is needed to assess in more detail, though, how different fiscal and healthcare systems across LMICs respond to economic instability, particularly in countries heavily dependent on foreign aid or with less robust private health insurance coverage.

## Conclusion

In Brazil, macroeconomic fluctuations significantly affect the fiscal space for health at the municipal level. While economic growth leads to increases in health and social spending, recessions negatively impact healthcare expenditures, particularly in vulnerable municipalities. Policymakers should prioritize protecting these critical areas of healthcare spending during economic downturns, with a focus on strengthening the resilience of local health systems to ensure they can continue providing essential services during periods of economic instability.

## Supplementary Material

czag043_Supplementary_Data

## Data Availability

The data underlying this article will be shared on reasonable request to the corresponding author.

## Reflexivity statement

This study was led by Brazilian researchers. We ensured gender balance with three male and two female authors and formed a multidisciplinary team from public health and economics. Three authors are based in Brazilian institutions (H.A., K.C., and R.R.), with two authors (T.H. and M.S.) from the Europe. H.A. and K.C. are early career researchers.
