# Supplementary material for: Macroeconomic fluctuations and the prioritization of healthcare funding by local governments: longitudinal evidence from 5461 Brazilian municipalities
Source: Health Policy Plan. 2026 Mar 30;41(5):887–97. doi: 10.1093/heapol/czag043 (PMC13187631; doi:10.1093/heapol/czag043)
Supplement: czag043_Supplementary_Data [file czag043_supplementary_data.zip › Appendix.docx]

**Additional Tables and Figures**

**Table A.1: Description of the outcome variables and data sources used**

| **Variable** | **Source** | **Description** |
| --- | --- | --- |
| Total revenue | Finbra | Total current revenue by municipality, divided by population to obtain per capita values and deflated using IPCA |
| Total expenditures | Finbra | Divided by population to obtain per capita values and deflated using IPCA |
| Health expenditures | SIOPS | Divided by population to obtain per capita values and deflated using IPCA |
| Education expenditures | Finbra | Divided by population to obtain per capita values and deflated using IPCA |
| Social expenditures | Finbra | Sum of expenditures on health, education, social assistance, transportation, urbanization, and culture. Divided by population to obtain per capita values and deflated using IPCA |
| Non-social expenditures | Finbra | Sum of all other expenditures (e.g., administrative, public safety). Divided by population to obtain per capita values and deflated using IPCA |
| Own-source health expenditures | SIOPS | Divided by population and deflated; health expenditures funded by municipal resources |
| Federal/State-financed expenditures | SIOPS | Calculated by subtracting own-source health expenditures from total health expenditures. It is the value of health expenditure financed by state and/or federal resources. Also divided by population to obtain per capita values and deflated using IPCA |
| Health Human Resource expenditures | SIOPS | Divided by population to obtain per capita values and deflated using IPCA |
| Health investment expenditures | SIOPS | Divided by population to obtain per capita values and deflated using IPCA |
| GDP per capita | IBGE | Municipal GDP divided by population and deflated using IPCA |
| Income per capita | Censo 2010 | Municipal average nominal monthly household income per capita |

**Note:** The table provides details about the outcome variables, including their source and how they were processed (e.g., per capita calculation, deflation, summation, or subtraction of variables).

**Table A.2: Changes in GDP and Revenue and Expenditures**

|  | Total Revenue per capita | Total Expenditures per capita | Social Exp. per capita | Non-social Exp. per capita | Health and Sanit Exp. per capita |
| --- | --- | --- | --- | --- | --- |
|  | (1) | (2) | (3) | (4) | (5) |
| GDP per capita in year t+2 | -0.0194∗ | -0.0021 | -0.0028 | 0.0056 | 0.0012 |
|  | (0.0107) | (0.0136) | (0.0183) | (0.0246) | (0.0386) |
| GDP per capita in year t+1 | 0.0111 | -0.0006 | -0.0071 | -0.0518∗∗ | 0.0183 |
|  | (0.0118) | (0.0104) | (0.0183) | (0.0238) | (0.0299) |
| GDP per capita | 0.0844∗∗∗ | 0.0612∗∗∗ | 0.0855∗∗∗ | 0.0737∗∗∗ | 0.1167∗∗∗ |
|  | (0.0154) | (0.0124) | (0.0152) | (0.0284) | (0.0312) |
| GDP per capita in year t-1 | 0.0214∗∗ | 0.0373∗∗∗ | 0.0377∗∗ | 0.0346∗ | 0.0249 |
|  | (0.0104) | (0.0105) | (0.0168) | (0.0197) | (0.0256) |
| GDP per capita in year t-2 | 0.0559∗∗∗ | 0.0452∗∗∗ | 0.0426∗∗∗ | 0.0554∗∗ | 0.0593∗∗ |
|  | (0.0100) | (0.0117) | (0.0142) | (0.0228) | (0.0261) |
| *Fixed-effects* |  |  |  |  |  |
| Municipality | Yes | Yes | Yes | Yes | Yes |
| Year | Yes | Yes | Yes | Yes | Yes |
| *Fit statistics* |  |  |  |  |  |
| R2 | 0.32304 | 0.34933 | 0.21349 | 0.15606 | 0.13435 |
| Observations | 76,437 | 76,437 | 76,437 | 76,437 | 76,437 |
| Dependent variable mean | 3,781.1 | 3,060.6 | 2,530.4 | 945.5 | 825.5 |

*Notes:* This table shows regression results following equation [1](#_bookmark0) for both contemporary GDP per capita effects as well as two leads and two lags to better understand dynamic effects over time. The dep. vars refer to Total Revenue, Total Expenditures, and categories of expenditures per capita. Standard errors clustered at the municipality level reported in parentheses. Abbreviation: GDP = Gross Domestic Product. Significance: ***p< 0.01, **p< 0.05, *p< 0.1.

**Table A.3: Changes in GDP and Health Expenditures**

|  | Health Exp.  per capita | HR Exp.  per capita | Investments Exp.  per capita |
| --- | --- | --- | --- |
|  | (1) | (2) | (3) |
| GDP per capita in year t+2 | 0.0126 | 0.0341 | 0.1087 |
|  | (0.0204) | (0.0576) | (0.1360) |
| GDP per capita in year t+1 | 0.0049 | -0.0701 | -0.0553 |
|  | (0.0138) | (0.0592) | (0.0914) |
| GDP per capita | 0.0605∗∗∗ | 0.1150∗∗∗ | 0.2838∗∗∗ |
|  | (0.0172) | (0.0369) | (0.0932) |
| GDP per capita in year t-1 | 0.0135 | -0.0210 | 0.1044 |
|  | (0.0154) | (0.0249) | (0.1466) |
| GDP per capita in year t-2 | 0.0716∗∗∗ | 0.0399∗∗ | 0.1633 |
|  | (0.0143) | (0.0182) | (0.1071) |
| Fixed-effects |  |  |  |
| Municipality | Yes | Yes | Yes |
| Year | Yes | Yes | Yes |
| R^2^ | 0.33344 | 0.18521 | 0.17483 |
| Observations | 76,437 | 76,437 | 76,437 |
| Dependent variable mean | 793.9 | 392.8 | 46.8 |

*Notes:* This table shows regression results following equation [1](#_bookmark0) for both contemporary GDP per capita effects as well as two leads and two lags to understand dynamic effects over time. The dep. vars refer to Total Health Expenditure and Health expenditure in HR and investments. Standard errors clustered at the municipality level reported in parentheses. Abbreviations: GDP = Gross Domestic Product; HR = Human Resources; Exp = Expenditures. Significance: ***p< 0.01, **p< 0.05, *p< 0.1.

Table (A.4) Recession and Revenue and Expenditures

Total Revenue Total Exp. Social Exp. Non-social Exp. Health Exp.

per capita per capita per capita per capita per capita

|  | (1) | (2) | (3) | (4) | (5) |
| --- | --- | --- | --- | --- | --- |
| Recession | -0.0058∗∗∗ | -0.0053∗∗∗ | -0.0070∗∗ | 0.0114 | -0.0153∗∗ |
|  | (0.0021) | (0.0019) | (0.0035) | (0.0123) | (0.0072) |
| *Fixed-effects* |  |  |  |  |  |
| Municipality | Yes | Yes | Yes | Yes | Yes |
| Year | Yes | Yes | Yes | Yes | Yes |
| *Fit statistics* |  |  |  |  |  |
| R2 | 0.31732 | 0.25429 | 0.21094 | 0.15461 | 0.13304 |
| Observations | 76,445 | 76,445 | 76,445 | 76,445 | 76,445 |
| Dependent variable mean | 3,781.1 | 3,060.6 | 2,530.4 | 945.5 | 825.5 |

*Notes:* This table shows regression follows an adaptation of equation [1](#_bookmark0) where we replace the continuous GDP variable. with a binary variable indicating the occurrence of a recession in a given year. The dep. vars refer to Total Revenue, Total Expenditures, and categories of expenditures per capita. Standard errors clustered at the municipality level reported in parentheses. Abbreviation: Exp = Expenditures. Significance: ***p< 0.01, **p< 0.05, *p< 0.1.

Table (A.5) Recession and Health Expenditures

|  | Health Exp.  per capita  (1) | HR Exp.  per capita  (2) | Investment Exp.  per capita  (3) |
| --- | --- | --- | --- |
| Recession | -0.0079 | -0.0152∗∗∗ | -0.0619∗∗ |
|  | (0.0049) | (0.0055) | (0.0299) |
| *Fixed-effects*  Municipality | Yes | Yes | Yes |
| Year | Yes | Yes | Yes |
| *Fit statistics*  R2 | 0.33080 | 0.18387 | 0.17476 |
| Observations | 76,445 | 76,445 | 76,445 |
| Dependent variable mean | 793.9 | 392.8 | 46.8 |

*Notes:* This table shows regression follows an adaptation of equation [1](#_bookmark0) where we replace the continuous GDP variable with a binary variable indicating the occurrence of a recession in a given year. The dep. vars refer to Total Health Expenditures, and Health expenditure in HR and investments. Standard errors clustered at the municipality level reported in parentheses. Abbreviations: GDP = Gross Domestic Product; HR = Human Resources; Exp = Expenditures. Significance: ***p< 0.01, **p< 0.05, *p< 0.1.

Table (A.6) Recession, Heterogeneity by Income per capita

Total Exp. Social Exp. Non Social Exp. Health Exp.

per capita per capita per capita per capita

|  | **below md** | **above md** | **below md** | **above md** | **below md** | **above md** | **below md** | **above md** |
| --- | --- | --- | --- | --- | --- | --- | --- | --- |
|  | (1) | (2) | (3) | (4) | (5) | (6) | (7) | (8) |
| Recession | -0.0107∗∗∗ | -0.0019 | -0.0153∗∗∗ | -0.0028 | -0.0070 | 0.0200 | -0.0221∗∗∗ | -0.0107 |
|  | (0.0025) | (0.0026) | (0.0042) | (0.0045) | (0.0047) | (0.0155) | (0.0061) | (0.0095) |
| *Fixed-effects*  Municipality | Yes | Yes | Yes | Yes | Yes | Yes | Yes | Yes |
| Year | Yes | Yes | Yes | Yes | Yes | Yes | Yes | Yes |
| *Fit statistics*  R2 | 0.23568 | 0.27316 | 0.24148 | 0.20086 | 0.14530 | 0.16763 | 0.14603 | 0.13081 |
| Observations | 38,203 | 38,242 | 38,203 | 38,242 | 38,203 | 38,242 | 38,203 | 38,242 |

*Notes:* This table shows regression follows an adaptation of equation [1](#_bookmark0) where we replace the continuous GDP variable with a binary variable indicating the occurrence of a recession in a given year. The dep. vars refer to Total Revenue, Total Expenditures, and categories of expenditures per capita. We split municipalities according to whether they are above or below the median income per capita in 2010 (approximately the middle of our sample). Standard errors clustered at the municipality level reported in parentheses. Abbreviation: Exp = Expenditures. Significance: ***p< 0.01, **p< 0.05, *p< 0.1.

Table (A.7) Recessions, Heterogeneity by Private Health Coverage

Total Exp. Social Exp. Non-Social Exp. Health Exp.

per capita per capita per capita per capita

|  | **below md** | **above md** | **below md** | **above md** | **below md** | **above md** | **below md** | **above md** |
| --- | --- | --- | --- | --- | --- | --- | --- | --- |
|  | (1) | (2) | (3) | (4) | (5) | (6) | (7) | (8) |
| Recession | -0.0129∗∗∗ | -0.0023 | -0.0151∗∗∗ | -0.0042 | -0.0077∗ | 0.0179 | -0.0182∗∗∗ | -0.0133 |
|  | (0.0024) | (0.0024) | (0.0033) | (0.0044) | (0.0045) | (0.0149) | (0.0053) | (0.0091) |
| *Fixed-effects*  Municipality | Yes | Yes | Yes | Yes | Yes | Yes | Yes | Yes |
| Year | Yes | Yes | Yes | Yes | Yes | Yes | Yes | Yes |
| *Fit statistics*  R2 | 0.24460 | 0.26313 | 0.27096 | 0.19756 | 0.14184 | 0.16577 | 0.13964 | 0.13425 |
| Observations | 37,318 | 39,127 | 37,318 | 39,127 | 37,318 | 39,127 | 37,318 | 39,127 |

*Notes:* This table shows regression follows an adaptation of equation [1](#_bookmark0) where we replace the continuous GDP variable with a binary variable indicating the occurrence of a recession in a given year. The dep. vars refer to Total Revenue, Total Expenditures, and categories of expenditures per capita. We split municipalities according to whether they are above or below the median private insurance coverage. Standard errors clustered at the municipality level reported in parentheses. Abbreviation: Exp = Expenditures. Significance: ***p< 0.01, **p< 0.05, *p< 0.1.

Table (A.8) Recessions, Heterogeneity by Health Spending with own resources

Total Exp. Social Exp. Non Social Exp. Health Exp.

per capita per capita per capita per capita

|  | **below md** | **above md** | **below md** | **above md** | **below md** | **above md** | **below md** | **above md** |
| --- | --- | --- | --- | --- | --- | --- | --- | --- |
|  | (1) | (2) | (3) | (4) | (5) | (6) | (7) | (8) |
| Recession | -0.0076∗∗∗ | -0.0035 | -0.0128∗∗ | -0.0017 | 0.0166 | 0.0051 | -0.0291∗∗ | -0.0039 |
|  | (0.0026) | (0.0026) | (0.0050) | (0.0041) | (0.0224) | (0.0063) | (0.0118) | (0.0064) |
| *Fixed-effects*  Municipality | Yes | Yes | Yes | Yes | Yes | Yes | Yes | Yes |
| Year | Yes | Yes | Yes | Yes | Yes | Yes | Yes | Yes |
| *Fit statistics*  R2 | 0.22747 | 0.28577 | 0.26030 | 0.18479 | 0.19990 | 0.13172 | 0.14467 | 0.12809 |
| Observations | 38,216 | 38,229 | 38,216 | 38,229 | 38,216 | 38,229 | 38,216 | 38,229 |

*Notes:* This table shows regression follows an adaptation of equation [1](#_bookmark0) where we replace the continuous GDP variable with a binary variable indicating the occurrence of a recession in a given year. The dep. vars refer to Total Revenue, Total Expenditures, and categories of expenditures per capita. We split municipalities according to whether they are above or below the median on the levels of municipal health spending with own resources, considering the average spending in our sample of years. Standard errors clustered at the municipality level reported in parentheses. Abbreviation: Exp = Expenditures. Significance: ***p< 0.01, **p< 0.05, *p< 0.1.

Figure (A.1) **Changes in GDP and Revenue and Expenditures, Heterogeneity by Recession and Boom**

**
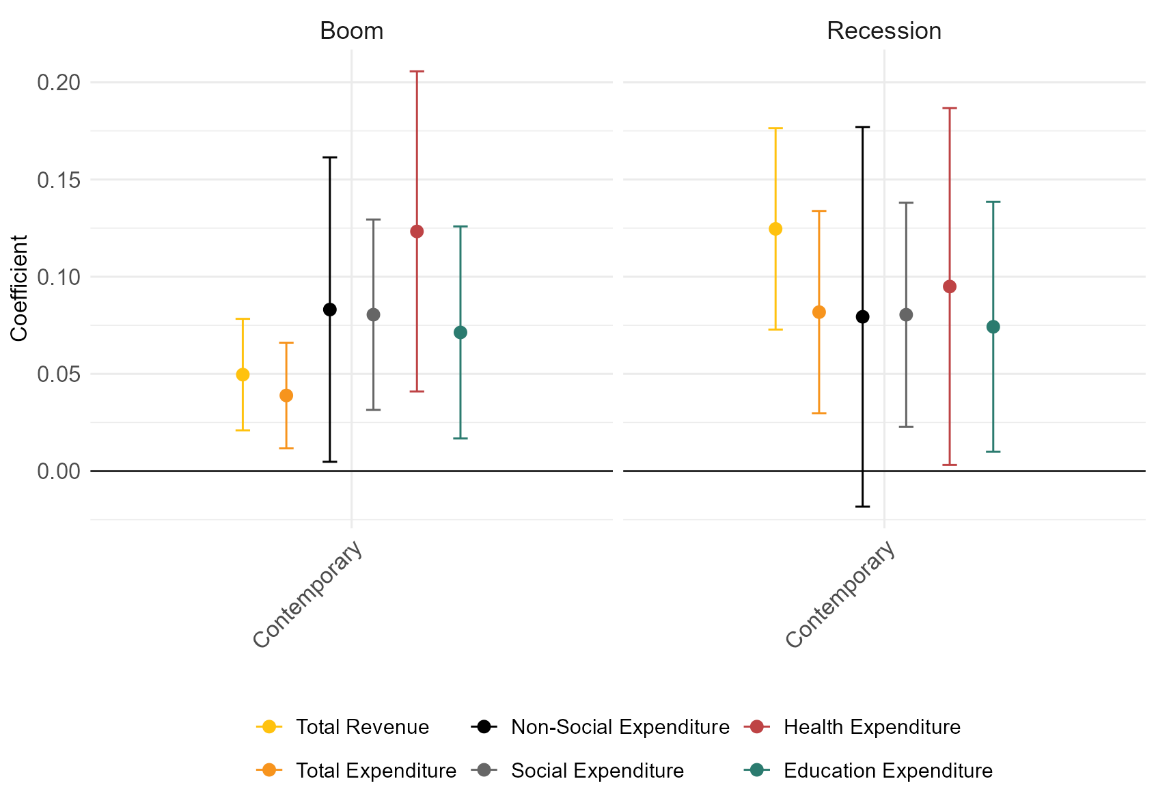
**

*Notes:* The estimates are based on equation 1, augmented with interactions between local GDP and indicators for economic booms and recessions. Reported coefficients correspond to the interaction terms, capturing regime-specific elasticities. We split municipalities according to whether they faced a recession or a boom in a given year. The dep. vars refer to Total Revenue, Total Expenditures, and categories of expenditures per capita. Standard errors clustered at the municipality level reported in parentheses. Abbreviations: GDP = Gross Domestic Product; HR = Human Resources; Exp = Expenditures. Significance: ***p< 0.01, **p< 0.05, *p< 0.1

Figure (A.2) **Changes in GDP and Health Expenditures, Heterogeneity by Recession and Boom**

**
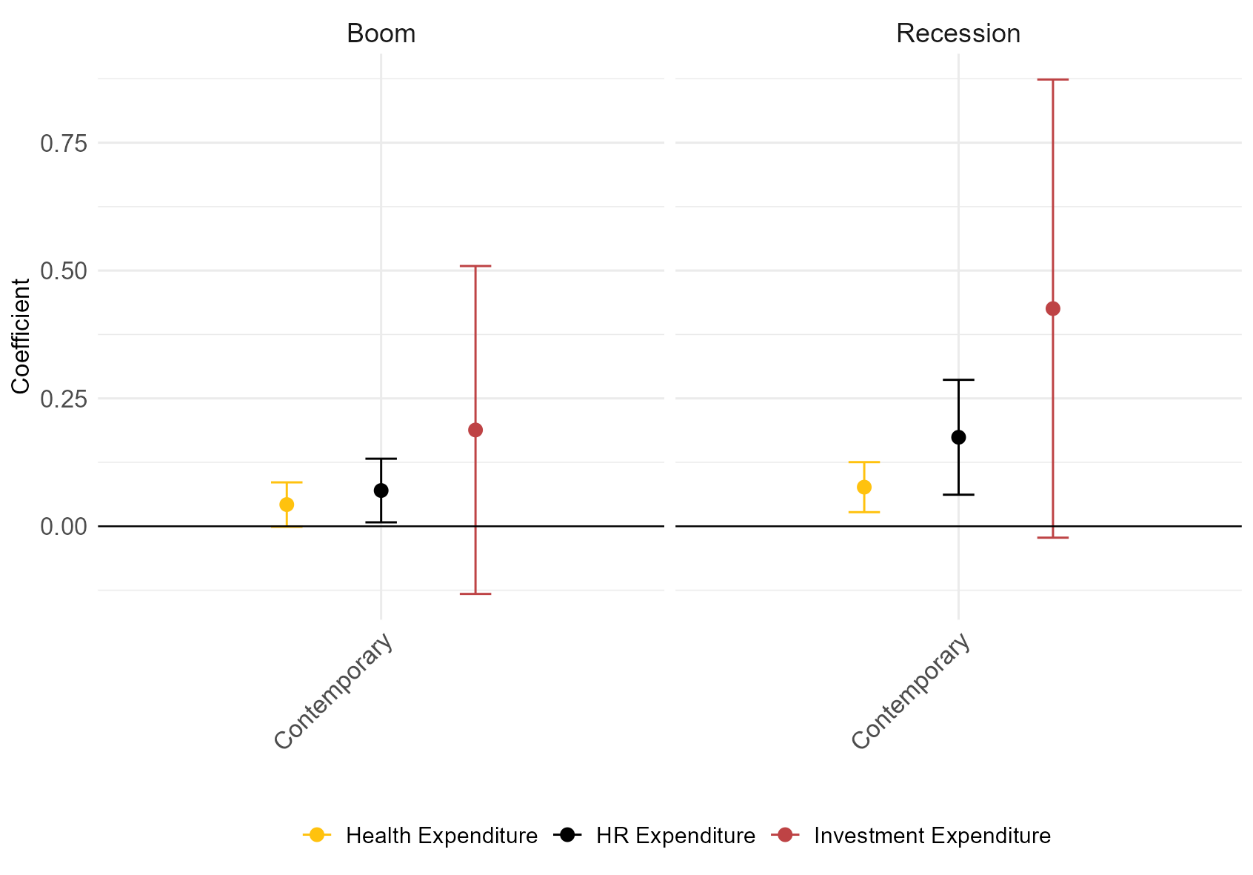
**

*Notes:* The estimates are based on equation 1, augmented with interactions between local GDP and indicators for economic booms and recessions. Reported coefficients correspond to the interaction terms, capturing regime-specific elasticities. We split municipalities according to whether they faced a recession or a boom in a given year. The dep. vars refer to Total Health Expenditures, and Health expenditure in HR and investments. Standard errors clustered at the municipality level reported in parentheses. Abbreviations: GDP = Gross Domestic Product; HR = Human Resources; Exp = Expenditures. Significance: ***p< 0.01, **p< 0.05, *p< 0.1
