# Supplementary material for: Macroeconomic fluctuations and the prioritization of healthcare funding by local governments: longitudinal evidence from 5461 Brazilian municipalities
Source: Health Policy Plan. 2026 Mar 30;41(5):887–97. doi: 10.1093/heapol/czag043 (PMC13187631; doi:10.1093/heapol/czag043)
Supplement: czag043_Supplementary_Data [file czag043_supplementary_data.zip › Table 1.docx]

**Table 1: Descriptive statistics of the budget variables used in the model**

|  | **Q1** | | **Median** | | **Mean** | | **Q3** | | **SD** | |  |
| --- | --- | --- | --- | --- | --- | --- | --- | --- | --- | --- | --- |
| **Municipal budgetary variables (R$ at 2022 prices, per capita)** |  | |  | |  | |  | |  | |  |
| GDP | 10,416.3 | | 18,075.2 | | 25,049.4 | | 30,436.5 | | 1,328,757.5 | |  |
| Change in GDP | -361.9 | | 507.5 | | 789.2 | | 1,649.0 | | 551,521.4 | |  |
| Income | 625.4 | | 1,024.2 | | 1,069.7 | | 1,396.4 | | 502.5 | |  |
| Total revenue | 2,498.9 | | 3,266.4 | | 3,781.1 | | 4,450.9 | | 2,079.7 | |  |
| Total expenditures | 2,070.0 | | 2,702.6 | | 3,060.6 | | 3,616.0 | | 1,555.3 | |  |
| Social expenditures | 1,747.9 | | 2,279.4 | | 2,530.4 | | 2,988.9 | | 1,510.4 | |  |
| Non-social expenditures | 519.2 | | 747.0 | | 945.5 | | 1,126.5 | | 803.0 | |  |
| Health expenditures | 518.5 | | 701.2 | | 793.9 | | 961.7 | | 412.8 | |  |
| Health expenditures - Municipal resources | 270.7 | | 414.7 | | 510.2 | | 645.8 | | 348.6 | |  |
| Health expenditures. - Federal/State resources | 179.7 | | 254.7 | | 284.8 | | 351.1 | | 169.7 | |  |
| Health HR expenditures | 245.6 | | 346.5 | | 392.8 | | 480.6 | | 224.9 | |  |
| Health investment expenditures | 10.9 | | 26.5 | | 46.8 | | 57.6 | | 65.6 | |  |
| **Other variables:** |  | |  | |  | |  | |  | |  |
| Relative change in GDP (%) | | -2.2 | | 3.9 | | 5.1 | | 10.4 | | 5,593.7 | |
| Population | | 5,341 | | 11,150 | | 34,681 | | 23,881 | | 12,099,887 | |
| Privately insured individuals (%) | | 0 | | 3.1 | | 7.6 | | 9.6 | | 10.9 | |

**Note:** The table provides five key statistical measures: the first quartile (Q1), median, mean, third quartile (Q3), and standard deviation (SD) for each budget category. Abbreviations: GDP = Gross Domestic Product; HR = Human Resources.
