# Supplementary figures and images for: Macroeconomic fluctuations and the prioritization of healthcare funding by local governments: longitudinal evidence from 5461 Brazilian municipalities
Source: Health Policy Plan. 2026 Mar 30;41(5):887–97. doi: 10.1093/heapol/czag043 (PMC13187631; doi:10.1093/heapol/czag043)

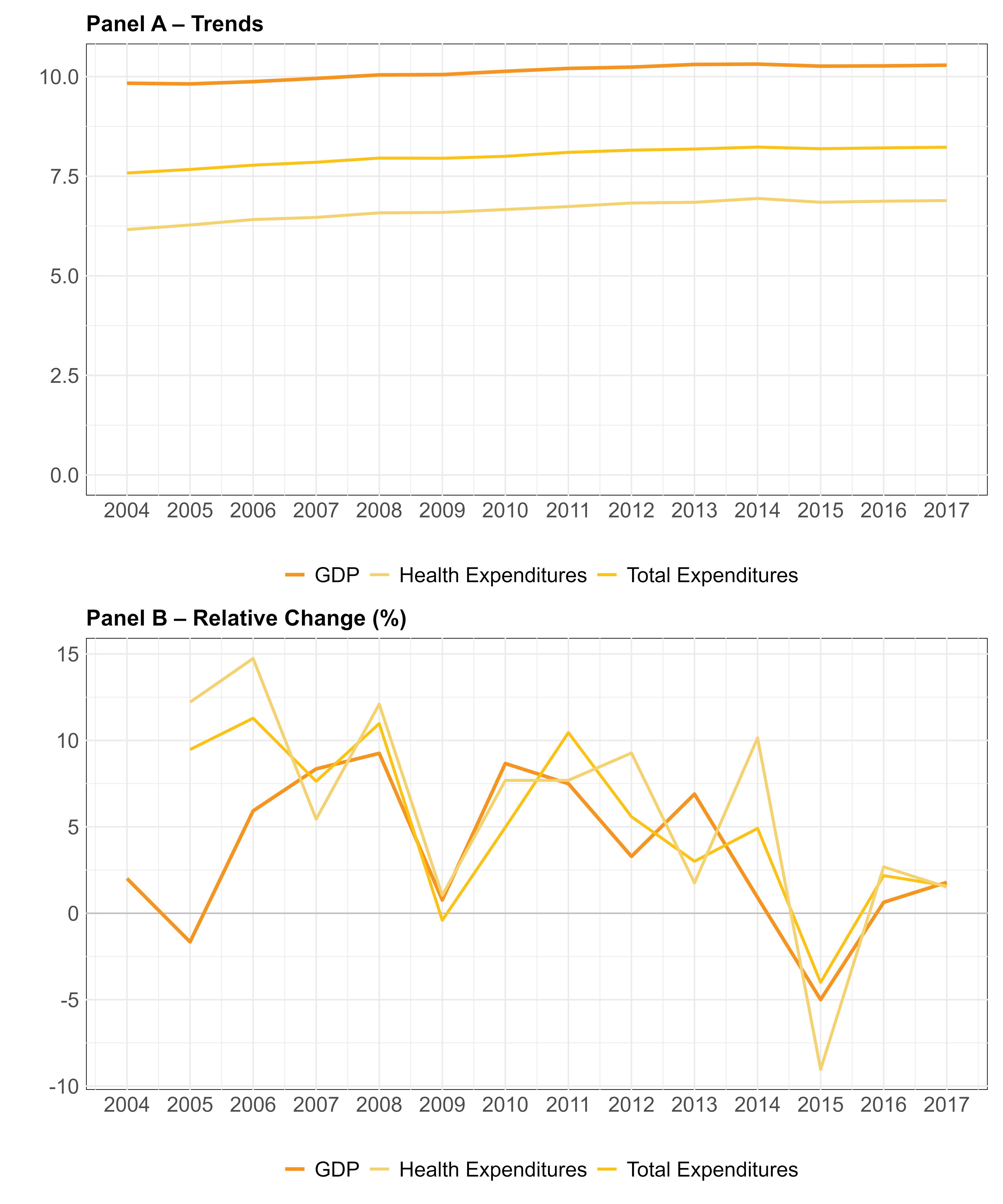

Supplement: czag043_Supplementary_Data [file czag043_supplementary_data.zip › Figure_1.tif]

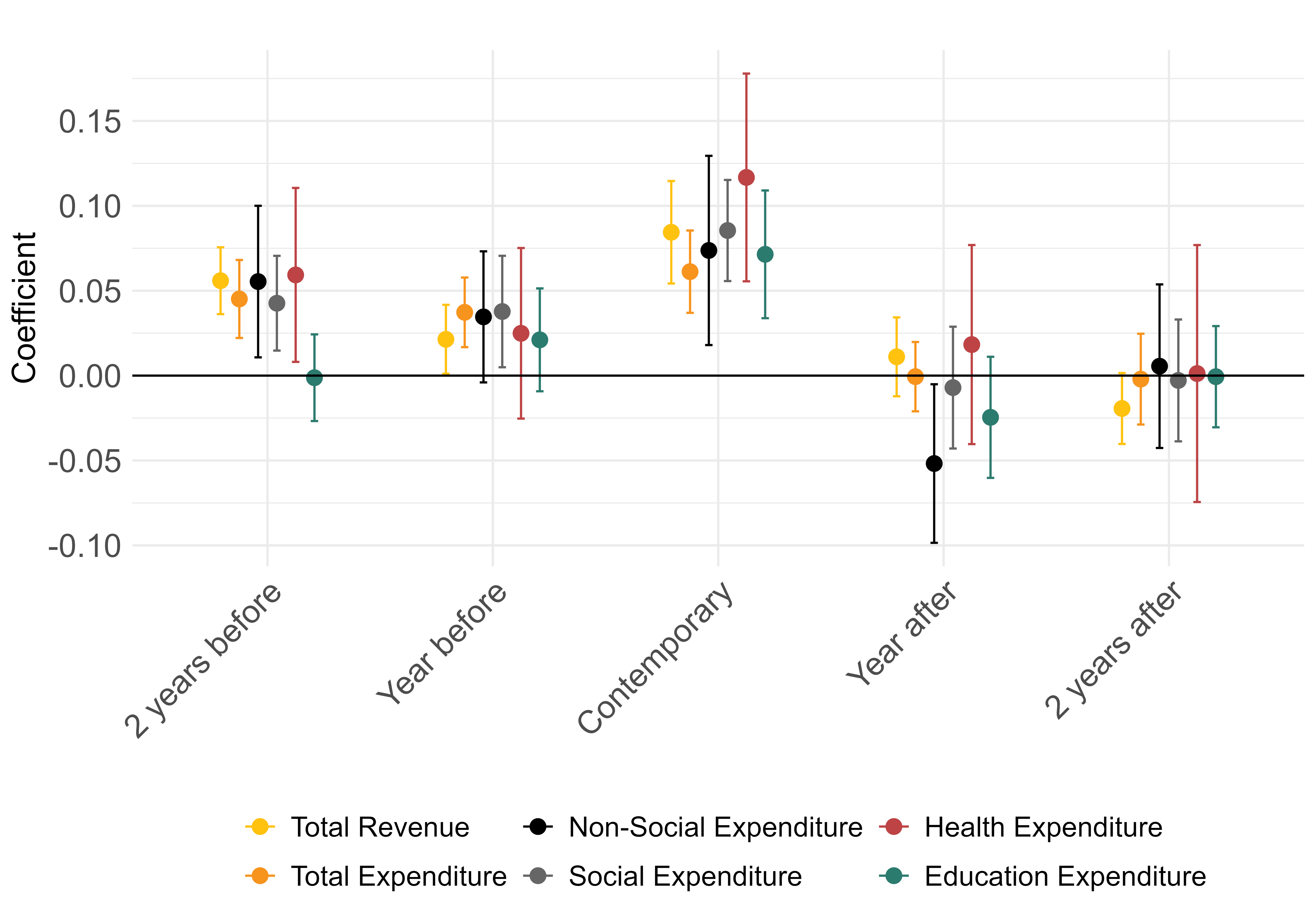

Supplement: czag043_Supplementary_Data [file czag043_supplementary_data.zip › figure_2.tif]

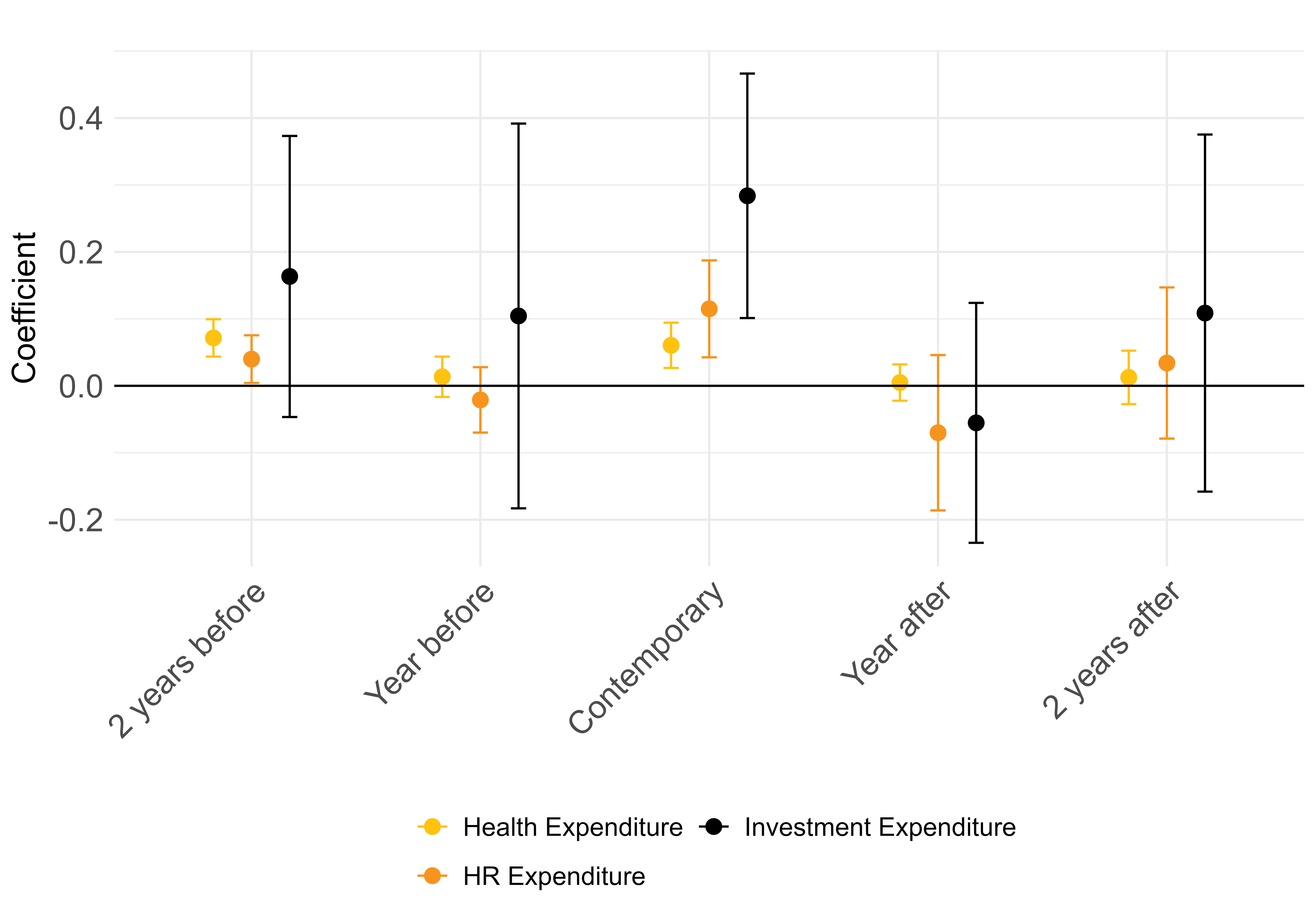

Supplement: czag043_Supplementary_Data [file czag043_supplementary_data.zip › figure_3.tif]

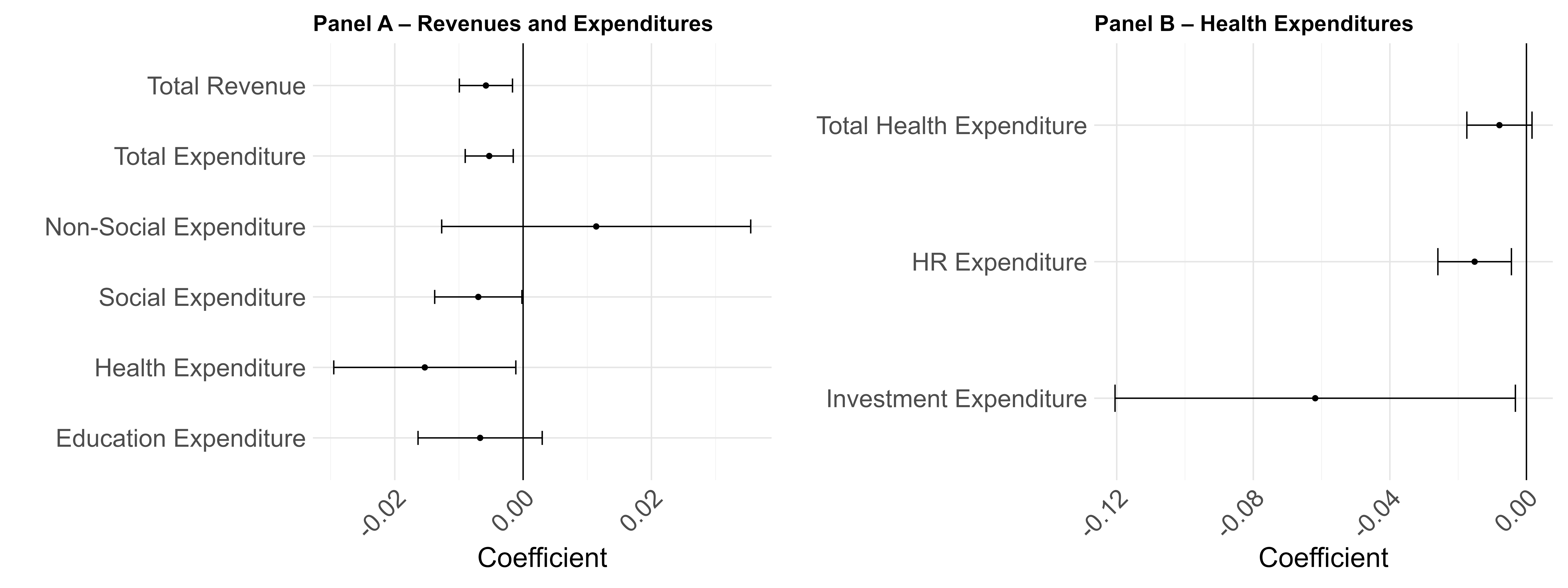

Supplement: czag043_Supplementary_Data [file czag043_supplementary_data.zip › Figure_4.tif]

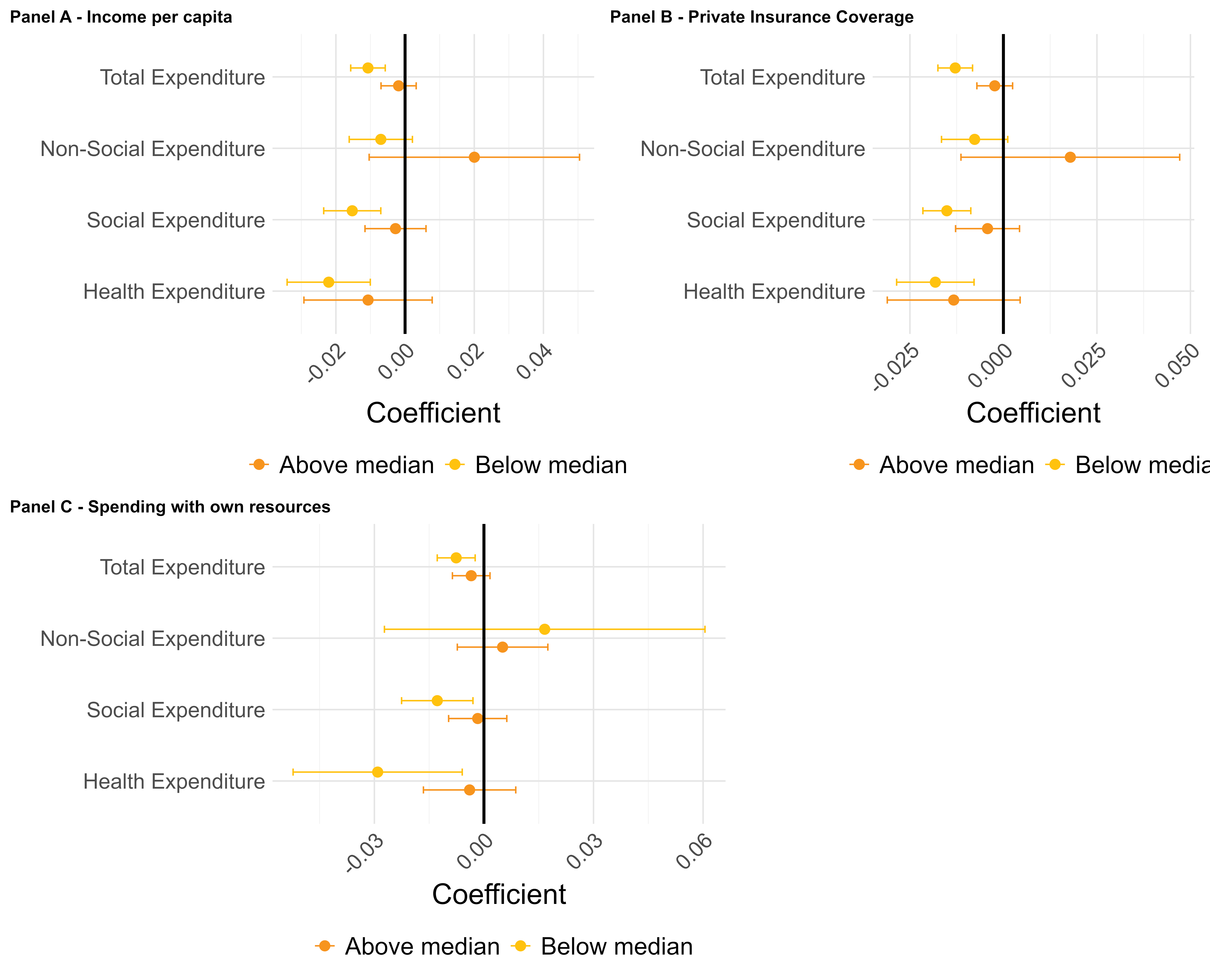

Supplement: czag043_Supplementary_Data [file czag043_supplementary_data.zip › Figure_5.tif]
